# Induction of Influenza (H5N8) Antibodies by Modified Vaccinia Virus Ankara H5N1 Vaccine

**DOI:** 10.3201/eid2106.150021

**Published:** 2015-06

**Authors:** Rory D. de Vries, Heidi L.M. De Gruyter, Theo M. Bestebroer, Mark Pronk, Ron A.M. Fouchier, Albert D.M.E. Osterhaus, Gerd Sutter, Joost H.C.M. Kreijtz, Guus F. Rimmelzwaan

**Affiliations:** Erasmus Medical Center, Rotterdam, the Netherlands (R.D. de Vries, H.L.M. De Gruyter, T.M. Bestebroer, M. Pronk, R.A.M. Fouchier, A.D.M.E. Osterhaus, J.H.C.M. Kreijtz, G.F. Rimmelzwaan);; Ludwig Maximilian University of Munich, Munich, Germany (G. Sutter)

**Keywords:** vaccine, avian influenza, MVA, clinical trial, antibodies, zoonoses, viruses

**To the Editor:** Aquatic birds form a natural reservoir of avian influenza viruses from which new human and animal influenza viruses originate. After initial detection in 2010 in China, a new highly pathogenic avian influenza (HPAI) virus of the H5N8 subtype reemerged in ducks in South Korea in 2014 (*1*,*2*). The hemagglutinin gene of this virus was distantly related to those of H5N1 subtypes that have caused infections in humans since 1997 (*3*). The World Health Organization/World Organisation for Animal Health/Food and Agriculture Organization of the United Nations H5N1 Evolution Working Group has assigned this new H5 to clade 2.3.4.4. Several poultry farms in the Netherlands, Germany, United Kingdom, and Italy were recently affected by infection with H5N8 virus closely related to the strains circulating in Asia (*4*), leading to implementation of preventive measures to restrict viral spread. Human infections with this new HPAI subtype have not been reported.

Modified vaccinia virus Ankara (MVA) is a promising viral vector platform for the development of influenza vaccines (*5*). We previously conducted a randomized double-blind phase 1/2a trial in young healthy persons to evaluate an MVA-based H5 vaccine (registered in the Netherlands’ trial register under NTR3401). Preclinical testing was conducted before this trial (*6**,**7*). Thirty-nine study participants received MVA-H5-serumfree Munich-Rotterdam (sfMR), which encoded hemagglutinin of influenza virus A/Vietnam/1194/2004 (H5N1), and 40 received vector control. Persons received 1 or 2 doses (with an interval of 4 weeks) of 10^7^ or 10^8^ PFU. Twenty-seven of the MVA-H5-sfMR–vaccinated persons received a booster vaccination 1 year later (again 10^7^ or 10^8^ PFU). The MVA-based vaccine was well tolerated and induced antibodies to both the homologous (A/Vietnam/1194/2004, clade 1) and a heterologous (A/Indonesia/5/2005, clade 2.1) H5N1 virus (*8*).

Although the newly emerged HPAI (H5N8) virus thus far has been detected only in birds, zoonotic transmission to humans exposed to large numbers of infected birds might occur (e.g., during culling operations). Therefore, shortly after the H5N8 outbreak in poultry in the Netherlands, we determined whether MVA-H5-sfMR–induced antibodies cross-react with the new H5N8 strain. Post-infection A/Vietnam/1194/2004 (clade 1) ferret serum (infected with a low pathogenic reverse genetics virus produced with hemagglutinin and neuraminidase gene segments of A/Vietnam/1194/2004 and the remaining 6 gene segments of A/Puerto Rico/8/34) was tested by hemagglutination-inhibition (HI) for cross-reactivity with viruses belonging to clade 0 (A/Hong Kong/156/1997), 2.1 (A/Indonesia/5/2005), 2.2 (A/Turkey/turkey/1/2005), and 2.3 (A/Anhui/1/2005) and the emerging H5N8 strain A/chicken/Netherlands/EMC-3/2014. A/Vietnam/1194/2004-specific serum (homologous titer 80) displayed low cross-reactivity with the clade 0, 2.2, and 2.3 viruses and completely failed to react with H5N8 strain A/chicken/Netherlands/EMC-3/2014. Inversely, A/chicken/Netherlands/EMC-3/2014-specific ferret serum (homologous titer 160) completely failed to cross-react with A/Vietnam/1194/2004. This finding demonstrates an antigenic distance between these viruses. Furthermore, the World Health Organization Collaborating Centers have only found limited cross-reactivity of a panel of H5 vaccine candidates with subtype H5N8 (*9*).

The clinical trial serum samples were pretreated with receptor-destroying enzyme and horse erythrocytes and tested by HI assay for their reactivity with A/chicken/Netherlands/EMC-3/2014 according to standard procedures (*10*). HI antibodies were induced after MVA-H5-sfMR vaccination that displayed considerable reactivity with the antigenically distinct H5N8 strain A/chicken/Netherlands/EMC-3/2014 (Figure, panel A). The titers of cross-reactive antibodies correlated with those to the homologous strain A/Vietnam/1194/2004 (*r* = 0.82, p<0.0001; Figure, panel B).

**Figure:**
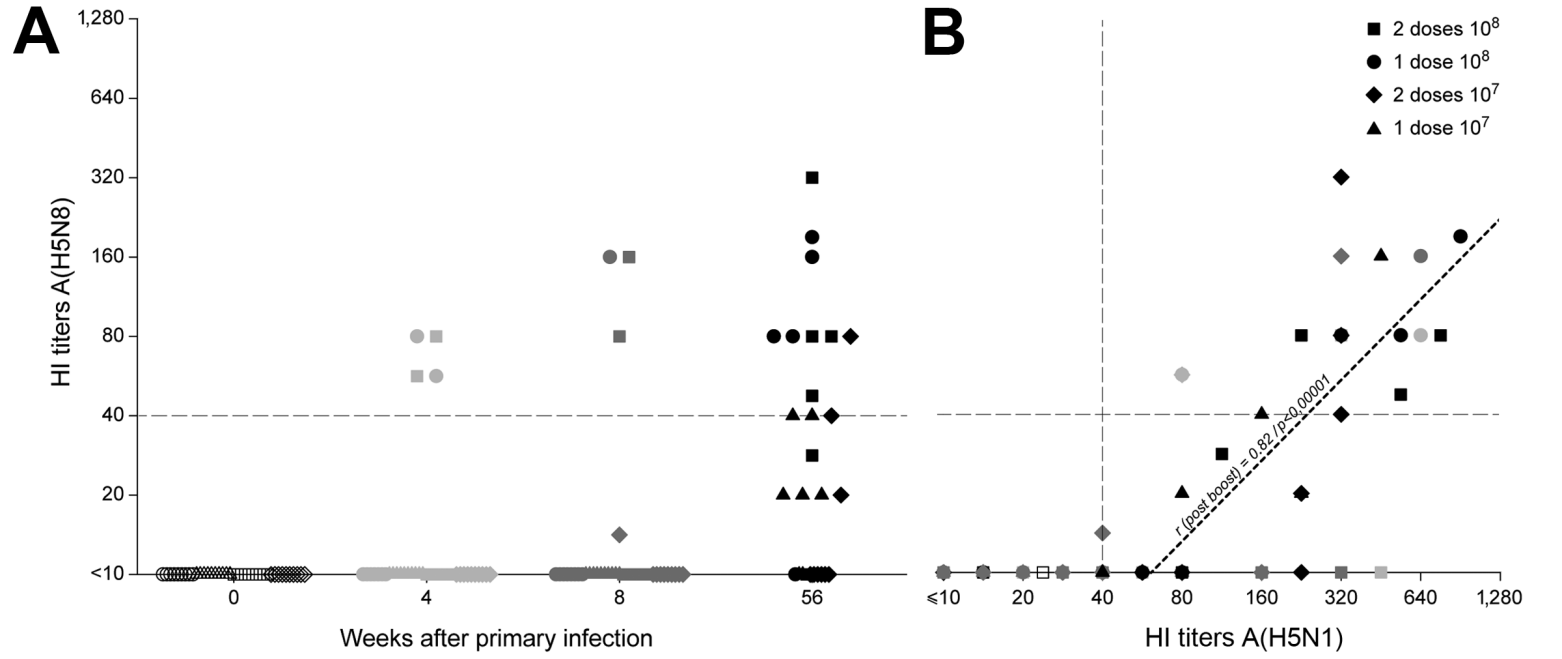
Results of hemagglutination-inhibition (HI) testing of modified vaccinia virus Ankara influenza vaccine cross-reactivity. Each symbol represents a person in the clinical trial; symbol shapes indicate different vaccination regimens. A) Timeline for development of HI titers against influenza virus A(H5N8) (A/chicken/Netherlands/EMC-3/2014). B) Correlation between HI titers against H5N8 and A/Vietnam/1194/2004 (H5N1) viruses. Linear regression for samples after booster vaccination is shown (*r* = 0.82, p<0.0001).

As shown previously (*8*), the magnitude of the antibody response was dose-dependent. Also, the highest cross-reactive response to the H5N8 strain was observed after vaccination with 10^8^ PFU (Figure, panel A) of MVA-H5. None of the study participants had prevaccination HI antibody titers >1:40 against A/Vietnam/1194/2004 or A/chicken/Netherlands/EMC-3/2014. Although most of the study participants had detectable HI antibody titers against the homologous virus 4 and 8 weeks after vaccination (*8*), antibodies against the H5N8 virus were barely detectable at these time points. HI antibody titers against the homologous virus increased in persons who received a booster vaccine at 52 weeks after primary vaccination. A large proportion (9 [82%] of 11 study participants; geometric mean titer 63) of participants who received a vaccine dose of 10^8^ PFU (equally divided among groups that received 1 or 2 previous doses) also had detectable cross-clade titers against the H5N8 virus A/chicken/Netherlands/EMC-3/2014. Furthermore, virus neutralizing antibodies against H5N8 virus were detected in 10 of 27 persons and correlated with antibody titers measured by HI assay.

We showed that an MVA-based H5 (A/Vietnam/1194/2004) vaccine can elicit cross-clade antibodies against the newly emerging HPAI (H5N8) virus that is genetically and antigenically distinct from the clade 1 H5N1 virus A/Vietnam/1194/2004. The cross-reactive antibody response observed after the 1-year booster vaccination suggests that the use of MVA-H5-sfMR is an effective emergency vaccination strategy in case tailor-made vaccines are not yet available in an outbreak situation. Thus, such a strategy might also be effective against the newly emerging influenza A(H5N8) viruses, in case these viruses would cause human infections.
